# Organizational impact in healthcare in France: a decade of insights but chicken and egg situation

**DOI:** 10.1017/S026646232610347X

**Published:** 2026-03-09

**Authors:** Tess Martin, Giovanny Arbelaez-Garces, Christophe Roussel, Nicolas Martelli, Olivier Marcellin

**Affiliations:** 1 Paris-Saclay University, GRADES, Faculty of Pharmacy, France; 2 Pharmacy Department, Georges Pompidou European Hospital, AP-HP, France; 3 Université de Strasbourg, CNRS, ICube, UMR 7357, France; 4 Edwards lifesciences, France; 5 Université Paris Dauphine-PSL, France

**Keywords:** organizational impact (OI), health technology assessment (HTA), medical devices (MDs), methodological frameworks, decision making

## Abstract

**Objectives:**

Over the past decade, organizational impact (OI) has gained recognition as a key dimension in health technology assessment (HTA) in France, particularly for medical devices. Despite the publication of a national framework by the Haute Autorité de Santé in 2020, the absence of standardized methodologies continues to hinder its integration into decision making.

**Methods:**

This commentary article traces the evolution of OI in French HTA, highlights real-world examples, and analyzes existing methodological tools, many adapted from other disciplines, that could enhance OI assessment.

**Results:**

It emphasizes the need for flexible, context-sensitive approaches and proposes recommendations to improve the robustness, reproducibility, and relevance of OI evaluations.

**Conclusions:**

The article also explores the implications for pricing and reimbursement decisions, as well as hospital-based HTA practices, aiming to support more structured and evidence-informed integration of organizational considerations into HTA processes in France and beyond.

## Introduction

Health technology assessment (HTA) has traditionally been defined as a multidisciplinary research field analyzing the medical, social, ethical, and economic implications of healthcare technologies. Initially focused on clinical and medico-economic evaluations, its scope has expanded to include organizational impact (OI), now considered essential in decision-making processes on health technologies (1; 2).

At the national level in France, the Haute Autorité de Santé (HAS), the French national HTA agency, has played a key role in this evolution, notably through the publication of an OI map for HTA in December 2020 (3). This guide offers a structured framework for assessing the OI of any health technology, including medicinal products, medical devices (MDs), and procedures. At the hospital level, HTA models developed through European programs like the AdHopHTA project (Adopting Hospital-Based HTA in the EU) reinforce this approach by considering organizational aspects alongside clinical effectiveness and safety (4). An international survey conducted in 2024 on HB-HTA practices further highlights the critical role of organizational factors, which are considered and analyzed in more than 60 percent of cases, underscoring their importance for local decision making (5).

MDs may disrupt healthcare workflows and infrastructures, making them intrinsically linked to OI. As early as 2015, French healthcare experts emphasized the need to consider OI, particularly in the context of MDs, which can exert a structuring influence on multiple dimensions of care organization (1). A 2025 systematic review further highlights that MDs frequently combine technological innovation with organizational process changes (e.g., service delivery, staffing, professional roles), making them inherently more complex to assess than traditional innovations (6). MDs will therefore serve as the guiding example throughout this commentary paper. Some, such as transcatheter aortic valve implantation (TAVI), directly impact surgical procedures and workforce management (7), while others, like remote monitoring technologies, necessitate major adaptations in patient care pathways (8). International institutions such as the OECD also emphasize that digital MDs impose significant constraints on workflows and infrastructures, requiring major adjustments in care pathways (9). In one of its 2025 guidelines, HAS notes: “In the case of medical telemonitoring, which most often corresponds to a follow-up modality that can transform certain care pathways or the organization of follow-up for chronic or acute conditions, the OI dimension can be extremely significant. However, HAS observes the difficulty in demonstrating this dimension, despite it being widely claimed in the dossiers submitted by manufacturers. Indeed, this aspect of the assessment is rarely documented and generally limited to descriptively reported elements” (10).

Despite growing awareness and the HAS guide on OI, the absence of a standardized methodology for assessing OI remains thus a major barrier – especially at the national level – hindering structured decision making for MD adoption and reimbursement. In response, this commentary paper aims to achieve three main objectives. First, it will trace the historical evolution of OI consideration in HTA in France, highlighting concrete examples. Second, it will analyze existing methodological tools – including those drawn from other disciplines – that could be adapted to improve OI assessment for MDs. Finally, it will propose structured recommendations aimed at strengthening current evaluation methodologies and ensuring their effective integration into healthcare decision making.

### Historical development of OI in HTA in France

The integration of organizational factors into HTA has progressively developed over the past decade in France. The need to consider OI beyond clinical and economic assessments was first emphasized during a collaborative think tank about health care (Ateliers de Giens (11)) in 2015, particularly for MDs. Roussel et al. *(1)* defined OI as “the assessment of the upstream and downstream consequences of introducing an MD in terms of resource allocation, production processes, availability, and information/training, depending on the perspective adopted,” explicitly excluding ethical, social, economic, legal, and sustainability-related factors.

These discussions laid the groundwork for a structured evaluation approach, which was formalized in the HAS OI map for HTA (2020) (3; 12). This framework identified three macrocriteria and corresponding subcriteria that could be affected by new medical products or practices: impacts on the care process (workflows, coordination, efficiency), impacts on stakeholder’ capabilities and skills (training and competencies required for implementation), and impacts on society and the community (accessibility, equity, and public health outcomes). By integrating organizational considerations into HTA, this initiative aimed to enhance decision making and provided a more holistic evaluation of medical innovations (12; 13).

When defining its methodology, HAS must comply with the laws and regulations set out in the French Social Security Code, within which OI can be assessed through two main aspects. Article R.165-2 of the French Social Security Code states that the Actual Clinical Benefit (ACB) incorporates an assessment of Public Health Interest (ISP) (14). Article R. 161-71-3 concerns the health economic evaluation conducted by HAS’s Economic and Public Health Evaluation Committee (CEESP) and identifies OI as a criterion triggering such an evaluation (15).

Furthermore, within the HTA process, eligibility for health-economic evaluation by HAS specifies that OI is required when the health product presents “a significant impact on national health insurance expenditures due to its effects on care organization, professional practices, or patient management pathways, and, where applicable, its price” (15). Additionally, the organizational relevance of an MD’s evolution is recognized as a legitimate reason for revising reimbursement conditions by the pricing authority in France, upon request from a company or representative body, as stated in Article 31 of the framework agreement between MDs manufacturers and the pricing authority from July 2024 (16).

Building on this framework, the quantification of OI has now become an essential aspect in the evaluation of MDs, and particularly for digital MDs (DMDs) (17). For instance, to be included on the list of medical telemonitoring activities, these technologies are assessed by HAS based on their expected contribution to medical care, considering their role within the patient management strategy according to three regulatory criteria:

1. Clinical improvement of the patient’s health status compared with conventional medical follow-up or, where applicable, with an already listed telemonitoring activity, taking into account adverse effects and risks associated with each mode of monitoring.
2. Significant gain in care organization, in terms of human and material resources and therapeutic treatments mobilized, without compromising the quality of care.
3. Public health interest, particularly regarding the expected impact on population health in terms of mortality, morbidity, quality of life, and the ability to address an unmet therapeutic need, considering disease severity and implications for health policies and programs (18).

This requirement reinforces the need for structured assessments based on the HAS OI map for HTA. It ensures that new digital health solutions contribute effectively to healthcare system efficiency and patient management.

However, the practical application of this OI remains a challenge. A recent case study attempted to use the HAS impact map to assess the organizational consequences of introducing immunotherapies for advanced cancers (19). While the study confirmed the relevance of the framework’s macrocriteria, it also highlighted key limitations in its implementation. In particular, the lack of methodological guidance on criteria selection, data collection, and result interpretation poses obstacles to its standardized application. Thus, while the HAS OI map provides a valuable structure for assessment, it does not function as a methodological tool. Instead, it serves as a reporting template, outlining broad evaluation criteria while leaving room for experts to determine the most appropriate methodological approaches for data collection and analysis within each specific HTA assessment. These findings underscore the need for further methodological refinement to ensure that OI assessments can be conducted consistently and meaningfully across different healthcare innovations.

In 2023, OI assessment saw further advancements, particularly regarding DMDs. During a collaborative think tank about health care, experts from academia, industry, and healthcare institutions emphasized the need to adapt evaluation frameworks to the specificities of DMDs (20). Given their rapid technological evolution, their integration into healthcare systems poses new challenges in terms of data security, interoperability, and real-world evidence generation. The discussions underscored the importance of developing clear methodological guidance for assessing the OI of these innovations. This led to calls for the HAS to provide dedicated recommendations and evaluation tools to better structure the assessment process.

At the same time, the French Association of Clinical Research Organization established a working group on the OI of MDs in hospital settings, focusing on how their adoption often requires significant structural and operational adjustments. While this initiative does not specifically target DMDs, it reflects a broader recognition of the need to integrate organizational considerations into HTA.

The increasing recognition of OI in HTA was further reflected in a 2024 national survey assessing access to innovative MDs across 60 French hospital centers (21). Among them, 90 percent (54/60) considered OI data collection essential, reinforcing the need for standardized methodologies and improved decision-making tools.

Several real-world case studies conducted in France in recent years highlight the importance of OI assessment for MDs, including the implementation of TAVI, a comparative study on single-use versus reusable bronchoscopes (22), and the application of an Organizational and Budget Impact Model to assess autotransfusion devices (23).

A recent initiative to incorporate OI data into French HTA submissions was seen in March 2025, when a manufacturer included real-world evidence in a reassessment dossier for an advanced wound dressing. The data focused on indicators such as the number of nurse visits, medical consultations, and dressing boxes used, linked to ulcer healing outcomes. While HAS acknowledged the relevance of these organizational aspects, it concluded that the data did not alter the appraisal of added value, due to methodological limitations – particularly the use of non-validated algorithms, and the presence of potential biases affecting interpretation (24).

Despite these efforts, the absence of official HAS guidelines on how to operationalize these assessments raises concerns about the acceptability, reproducibility, and standardization of OI evaluations, especially in complex healthcare settings. To address these limitations, existing methodological tools from other disciplines should be explored and adapted to optimize OI assessment and strengthen its integration into HTA and its impact on pricing decisions. The next section will examine potential methodologies to improve the robustness and applicability of OI assessments, ensuring a more structured and reproducible approach to decision making in HTA.

### Overview of common methods to assess OI

To respond effectively to the challenges posed by HTAs with their innovative features, such as those of DMDs, the question arises of appropriate methodological choices “offering a solid foundation” to guide their new integration into medical practices (20). The methodological choices for the evaluation of the OI of these devices must be adaptable and sufficiently flexible. This position is justified by the heterogeneity of health technologies and the types of care organizations in which they are inserted, which requires a combination of different approaches to be defined according to the evaluation objectives and the desired methodological rigor. The same is true for regulatory and ethical aspects, because when these studies take place in a hospital setting, constraints on the collection of patient data lead to decision-making choices for the design of the studies. In addition, the research objective also sets these directions depending on whether the expected outcome of the OI assessment is to observe it, or to understand the causal effects of each OI, or to explain it, and also, whether the organizational indicators have an impact on the health economic aspects. Finally, it is a question of guaranteeing the reproducibility and validity of the results according to the organizational contexts in which the OI evaluations are carried out, which also calls for tailor-made approaches and models, including appropriate triangulation.

For a given type of MD to be evaluated, defining the most relevant OI criteria and indicators depends on the hypotheses regarding the specific improvements expected in the patient pathway. By clearly formulating the hypotheses and using evidence from the literature, the evaluation can remain focused on the most critical outcomes (20) according to the following approach: choosing a conceptual framework to identify how the MD to be evaluated is expected to produce its effects and then aligning the identified criteria and indicators with the expected or observed effects. For example, if the device aims to reduce manual entry of medical data, changes in administrative burden or error rates can be measured using real-world data, including claims data, medical-administrative registers, medical registries, or other relevant sources. To achieve this, techniques derived in particular from the human and social sciences such as the construction of a map of care processes, expert opinions via individual interviews, focus groups with health professionals, or Delphi approaches, help in the choice and then validation of the OI criteria and indicators to meet this first milestone of HTA (25; 26). More broadly, it may be a question of mobilizing models to represent the organization, current states and the future, to generate alternative impact scenarios (27). For this purpose, computer science and probabilistic statistics can be used to model the care pathway for patients using databases, to provide interpretable dynamic indicators and thus provide a solid basis for simulation models or the prediction of future impacts (28–30).

Regarding primary data collection, we can start by clarifying the objectives and expectations of the study, as well as considering potential biases. For example, the risks of contamination between groups in with-and-without study designs or the difficulty of interpreting the temporal trend in before–after designs, which must be adjusted accordingly. For analysis, the propensity score matching (31) method or inverse probability of treatment weighting (32) can be used to minimize selection bias in the retrospectively analyzed data, thus creating a “pseudo-population” closer to a randomized trial. In addition, the difference-in-differences method allows controlling for unobserved confounders that remain stable over time (33). When it comes to explaining causal mechanisms (34) for the interpretation of results, additional qualitative techniques can be added to the data collection, such as process tracing (or tracing underlying mechanisms) using qualitative questionnaires addressed to patients and professionals. In the same way, a structured approach such as the “patient tracer” technique proposed by the HAS in another context to assess the quality of care pathways – which can be used to evaluate how multiprofessional and multidisciplinary patient care aligns with established standards of practice – can also be mobilized here for the OI (35; 36). This helps reveal how and why the introduction of new technology leads to specific organizational changes by examining the intermediate stages (19). Also, with a view to explaining the effects, the realistic evaluation approach focuses on what works by emphasizing the interaction between context, mechanism, and outcome. Again, qualitative approaches such as individual interviews, focus groups, or Delphi approaches, can be used at this stage to identify the obstacles and factors promoting organizational change, while content analysis extracts explanatory factors from users’ discourse for interpretation work (37). Value stream mapping (or process mapping) is another explanatory tool used in change management to clarify workflows and to identify stages with high potential for organizational disruption (38), that can be used in HTA studies (39; 40). Finally, data-driven approaches such as microsimulation allow for the estimation of potential OIs by modeling different scenarios and exploring long-term effects beyond the immediate study period (41; 42).

The advantage of hybrid approaches is to calibrate simulation models with real data in order to extrapolate the results to predict future performance under various hypotheses (35). Therefore, by integrating and combining these tools and techniques borrowed from other fields of research, such as public health, health system sciences, or health related social sciences among other, it is possible to develop robust and adaptable methodologies for OI assessments of MDs that meet the new requirements posed by the evolution of health technologies in healthcare organizations.

A summary of the methods and tools discussed in this section – along with their respective advantages, limitations, and relevance for assessing OI – is provided in Table 1 (it is nevertheless not an exhaustive description of them). This overview is intended as a reference for evaluators and practitioners, enabling them to select an appropriate combination of approaches and techniques tailored to their specific evaluation context and objectives. By doing so, they can construct methodologically sound OI assessments that are aligned with both the nature of the MD under evaluation and the characteristics of the healthcare organization involved.

**Table 1.** Overview of methods and tools for assessing organizational impact

Study design	Study type	Description	Main limitations	Risk level	Type of approach	Relevance for OI assessment	Example of references using the method
Observational	Cross-sectional	Observation of an organization at a point in time.	No evolution or causality, risk of selection bias.	None	Quantitative, qualitative or mixed	State of the art, one-off comparison	(47)
Observational	Longitudinal	Repeated monitoring of a system over time.	Costly, loss to follow-up, complex analysis.	Variable	Mixed	Organizational evolution	(48)
Observational	Prospective cohort	Monitoring of a group exposed/not exposed to a technology, prospective data collection.	Long and costly.	Low to moderate	Quantitative, sometimes qualitative	Impact measurement and causality	(49)
Observational	Retrospective cohort	Retrospective analysis of existing data.	Variable data quality.	None	Quantitative, sometimes qualitative	Variable relevance depending on data quality	(50)
Quasi-experimental	Cross-sectional before–after, without control group	Before/after comparison in the same department.	Limited causal attribution.	None	Mixed	Evaluation of global change	(51)
Quasi-experimental	Before–after with control group (difference-in-difference)	Comparison of changes in two departments (exposed vs. unexposed).	Complexity, selection bias.	Low to moderate	Mixed	Isolates the effect of innovation	(52)
Quasi-experimental	Interrupted time series (ITS)	Repeated measurements before/after intervention.	Requires many measures.	Moderate	Quantitative (segmented regression), sometimes mixed	Evaluation in real context	(53; 54)
Quasi-experimental	With-without intraservice	Comparison of service over two periods (without/with intervention).	Sensitive to other changes.	None	Mixed	Impact of an internal reorganization	(55; 56)
Quasi-experimental	With-without intraservice in cluster	Comparison of subunits exposed or not to an intervention.	Risk of contamination, complex logistics.	Present	Mixed	Evaluation at department level	(57)
Quasi-experimental	With-without interservice in cluster	Comparison between exposed and unexposed departments.	Selection bias, initial differences.	Low to moderate	Mixed	Comparison and generalization	(51)
Experimental	Randomized controlled trial (RCT)	Randomization of departments or units, pre-post follow-up.	Costly, ethical limits.	Low	Quantitative	Relevant	(58)
Experimental	Randomized controlled trial (RCT) in cluster	Randomization by groups (departments, teams).	Complex, labor intensive.	Low	Quantitative	Relevant for collective interventions	(59)

*Note:* This table summarizes the key approaches discussed, highlighting their respective study type, advantages, limitations, and relevance for evaluating the impact of medical devices within healthcare organizations. It serves as a practical reference to guide evaluators in selecting appropriate methodologies tailored to specific contexts and objectives.

### Recommendations and perspectives for improvement

The current methodological approaches identified for assessing OI in HTA exhibit significant limitations. In particular, they often lack the necessary specificity and adaptability required for the evaluation of diverse healthcare technologies, and they are rarely standardized across institutions. These gaps hinder the integration of OI into decision-making processes, both at the national and local levels. Moving forward, a more structured and collaborative methodological effort is needed to ensure that OI assessments are reliable, reproducible, and decision relevant.

At the national level, the evolution of existing HTA frameworks should be considered a priority. First, it is essential that existing evaluation guides, such as those provided by the HAS, incorporate both qualitative and quantitative methodological tools specifically designed to measure OI, as is already the case, for example, in the HAS evaluation guide dedicated to early access drugs (43). These tools should enable evaluators to capture the diversity of impacts, from workflow reorganization to changes in training requirements, care coordination, or resource allocation. A formal commitment from HAS to lead this methodological development is needed with the support of several agencies, including the French Health Innovation Agency (Agence de l’Innovation en Santé) (44). This could include the establishment of a dedicated multidisciplinary working group, a structured consultation process involving clinical and methodological experts, and a phase of public feedback to promote transparency and legitimacy.

As previously noted, HAS assesses OI through two regulatory dimensions: ACB and the health-economic evaluation conducted by the CEESP. Although economic evaluation incorporates OI, most MDs are not subject to economic assessment by CEESP and are evaluated solely on clinical grounds. In addition, HAS guidance on MD evaluation refers to OI only indirectly, within the context of clinical added value (CAV) (10). It is unclear whether OI alone can influence an increase in CAV level, particularly when clinical outcomes are equivalent across competing technologies, or how it affects pricing decisions. Clarifying the weight and status of OI within the HTA and the pricing processes would strengthen the consistency and transparency of decisions. Such clarifications may require regulatory adjustments, given that the modalities for evaluating MDs by the HAS are currently embedded in the French Social Security Code.

At the local level, the integration of OI into HB-HTA processes remains poorly structured. Evidence from initiatives such as the European AdHopHTA project (45) and a French survey on HB-HTA (21) has shown that OI is either absent or only implicitly addressed in many local evaluation protocols. However, as previously underscored in this commentary paper, a recent survey published in 2025 on international HB-HTA practices confirms that organizational aspects remain a key consideration for hospitals engaged in HB-HTA activities (5). This heterogeneity reflects a broader lack of shared methodological standards across institutions. To address this gap, it is crucial to develop national or regional guidelines that explicitly include OI as a core criterion in local HB-HTA processes. Such guidelines would support hospitals in structuring their evaluations, improving the comparability of results, and facilitating the adoption of technologies that offer genuine organizational improvements.

In parallel, real-world experimentation should be encouraged to validate and refine these methodological approaches. Pilot projects could be developed in settings where the introduction of a new MD has a clear and measurable OI. For example, devices that require coordination among multiple clinical teams, or those used in complex care pathways for specific diseases, present ideal conditions for methodological testing. Similarly, expert centers with experience in local HTA could serve as test beds for evaluating OI methodologies in diverse organizational contexts. These field studies would provide valuable empirical insights and support the generalization of best practices.

## Conclusion

The assessment of OI has emerged as a key component in the evaluation of health technologies within HTA processes. As health technologies become increasingly complex and embedded in clinical workflows, understanding their organizational consequences is essential to ensure safe, efficient, and sustainable implementation. While frameworks such as the HAS OI map provide valuable guidance, they fall short of offering fully operational tools that can be consistently applied in real-world settings.

To move beyond conceptual frameworks and toward practical integration, there is a pressing need for a structured and flexible methodological approach to OI assessment. Such an approach must be capable of addressing the diversity of healthcare technologies and organizational configurations, and it must be grounded in robust data collection, rigorous analysis, and interdisciplinary collaboration. By combining tools from qualitative research, social sciences, epidemiology, and systems modeling, evaluators can develop adaptable strategies that reflect both the realities of clinical practice and the requirements of evidence-based policy making.

Finally, the challenges associated with OI assessment are not unique to France. Other European countries face similar difficulties, and the forthcoming implementation of the Health HTA regulation provides a timely opportunity for convergence (46). Efforts should be made to develop a common definition of OI and to harmonize evaluation methodologies across EU Member States. This would strengthen the comparability of HTA results and support coordinated decision making on the adoption of innovative medical technologies throughout Europe.
